# Hereditary Taints

**Published:** 1879-04

**Authors:** Ephraim Cutter

**Affiliations:** Boston; Chicago


CHICAGO MEDICAL

### JOURNAL & EXAMINER.

VOL. XXXVIII.—APRIL, 1879.—No. 4.

[In these pages dimension and weight are expressed in terms of the Metric System, and temperature in degrees of the Centigrade Scale.]

### Original Lectures.

### ARTICLE I.

HEREDITARY TAINTS. An illustrated lecture delivered before the Chicago Medical Society, Feb. 17th, 1879, by EPHRAIM CUTTER, M.D., Boston.

"*Nothing taints sound lungs sooner than inspiring the breath of consumptive lungs.*"—*Harvey.*

Hereditary traits are those which are transmitted from parent to child. They may be good or bad. The latter are called taints (Greek, *teggo*, to wet: Latin, *tingere*, to dye or tincture.) To-day the subject involves heredity, which also includes physical, psychological, civil, social, political and monetary abilities and disabilities.

In compliance with your expressed desire to see what I have to show and to hear what I have to say, I come as a private from the picket line to report to his superiors—didactically, not polemically. "To prevent rash misconceptions and uncandid reflections that are always apt to arise from a partial view of any subject," allow me to say that I am prepared to be challenged, and to encounter opposite opinions—that I desire to be respectful to all—that I honor an honest, gentlemanly, though severe critic, but any discourteous treatment of my subject I regard as beneath the dignity of science; that my presentation of the subject is the fruit of years of toil and investigation, and is what I honestly believe it is here represented to be. If shown to be mistaken, I am ready to apologize, but I will not ask pardon for acting up to my convictions. Time alone can decide the questions raised.

In his voluminous, painstaking and authoritative work, Dr. Lionel Beale has extensively promulgated his idea respecting an hereditary taint, teaching that the primitive, formative, animal vital matter called by Mohl protoplasm (*proto*, first; *plasm*, form, mould or substance), and by him (Beale) called bioplasm, (*bio*, life; plasm), (Dr. L. Elsberg calls it better, bioplasson, life *forming*, instead of form)—has a degenerative or lowered type impressed upon it so that it develops what we call disease. In other words, that the taint consists of bioplasm running riot under mob law, breaking through the bodily systemic restraint that governs the various minor organisms of substance, which, when combined in a well-ordered and systematic colony we call a healthy human being. Dr. Beale has rendered a great service to mankind by liberating us from the thralldom of the cell doctrine. The greatness, number and value of his contributions to micrology (*micros*, small; *logos*, account), have made him famous. If I understand him rightly, cancer, syphilis and consumption—to name no more examples—are hereditary in that the dyed or tinged, or tinctured bioplasm in the parent is transmitted to the child—that it is not any morphologically distinctive thing, but is only discernable through the ruin wrought by its development—as seedsmen tell seeds by cultivation. If my opinion were asked, I would say that this doctrine is admirable for the cancerous diathesis, but as far as concerns the other diseases named, there is another man who, in 1868, propounded another doctrine—incidentally, as a matter of history, not intending to conflict with nor derogate from any other theorist. In the *American Journal of the Medical Sciences* for Jan., 1868, page 19, line 12, occur these words: (the title of the paper is "New Algoid Vegetations in Syphilis, etc.")—"This vegetation may be transmitted from one individual to another during the secondary and tertiary stages under proper conditions, without producing the primary disease. I have noticed many instances in which, the father having had the disease previous to marriage, and when the poison was not entirely eliminated, even though no outward manifestation of the disease had shown itself in him after marriage, this vegetation was transmitted to, and found in the blood of the wife and children many years after. In many cases of this kind, this vegetation produces no visible impression upon the systems to which it is transferred, while upon others it produces more or less marked constitutional disturbance." That is, the vegetation found in the blood of the parent is found in the blood of the child, and thus we have an hereditary taint which is a physical substance capable of being seen, the seeds, so to speak, of which are smaller than the red corpuscles, and therefore capable of infecting the child through the medium of the blood, it being an evident mechanical proposition that as the blood corpuscles of the mother are freely transmitted to the fœtal circulation, then any bodies smaller than the red corpuscles floating in the maternal blood will of necessity be readily found in the blood of the embryo, or, to put it differently, the seeds of the vegetation are the seeds of the disease, and find their way into the blood of the fœtus through the placenta.

It is my design to throw upon the screen some physical evidence of this statement. A ten years experience is my warrant for presenting it. I have found this evidence of practical value. Quietly I have been able to settle in my own mind, without using words, the diagnosis of syphilis, and to institute treatment accordingly, which the result has proved to be correct. Were this a matter of scientific interest alone, I should not feel justified in staking my professional character and reputation by appearing before you with my present purpose. I am only following in the steps of my great master, "whose shoe-latchet I am unworthy to loose." Who is this master? The following is a list of his works up to July, 1878: there are 42 papers and books, and 20 unpublished works and papers. I am not competent to express an opinion as to all, but I do know that some of them show a clearer insight into the nature of our common and most fatal diseases than I have found elsewhere. I respectfully ask a careful perusal of this list, should copies of it come into your possession.

PUBLISHED WORKS AND PAPERS OF J. H. SALISBURY, A.M., M.D.

1. Analysis of Fruits, Vegetables and Grains. N. Y. State Geological Reports. 1847-48-49.

2. PRIZE ESSAY.—Chemical Investigation of the Maize Plant in its various stages of growth, with the temperature of the soil at various depths, and that of trees in different seasons of the year. 206 pages. State Agricultural Reports of N. Y. and Ohio. 1849.

3. Chemical Analysis of Five Varieties of the Cabbage. 1850.

4. Rheum Rhaponticum. Chemical examination of the various parts of the plant. 1850.

5. Chemical Examination of Rumex crispus. 1855.

6. Experiments and Observations on the Influence of Poisons and Medicinal Agents upon Plants. 1851.

7. Chemical Examination of the Fruit of five (5) varieties of Apples. 1850.

8. Chemical Investigations connected with the Tomato, the Fruit of the Egg Plant, and Pods of the Okra. 1851.

9. History, Culture and Composition of Apium graveolens and Cichorium intibus. 1851.

10. Some Facts and Remarks on the Indigestibility of Food. 1852.

11. Compositions of Grains, Vegetables and Fruits. Ohio State Agricultural Reports. 1861.

12. Microscopic Researches, resulting in the discovery of what appears to be the cause of the so-called "Blight" in Apple, Pear and Quince Trees, and the decay in their fruit: and the discovery of the cause of the so-called "*Blister and Curl*" in the leaves of Peach Trees; with some observations on the development of the Peach Fungus. Illustrated with 6 plates. Ohio State Agricultural Reports. 1863.

13. Chronic Diarrhœa and its Complications, or the diseases arising in Armies from a too exclusive use of Amylaceous Food, with interesting matter relating to the Diet and Treatment of these abnormal conditions, and a new Army Ration proposed with which this large class of diseases may be avoided. The Ohio Surgeon General's Report for 1864.

14. Something about Cryptogams, Fermentation and Disease. St. Louis *Medical Reporter*, February, 1869.

15. Probable Source of the Steatozoon folliculorum. St. Louis *Medical Reporter*. January, 1869.

16. Investigations, Chemical and Microscopical, resulting in what appears to be the Discovery of a New Function of the Spleen and Mesenteric and Lymphatic Glands. Do. August, 1867. 29 pages.

17. Defective Alimentation a Primary Cause of Disease. Do. March and April 1 and 15, 1868. 70 pages and two plates of illustrations.

18. On the Cause of Intermittent and Remittent Fevers, with Investigations which tend to prove that these affections are caused by certain species of Palmellæ. *American Journal Medical Sciences*, 1866. Also in *Revue Scientifique*. November, 1869.

19. Some Experiments on Poisoning with the Vegetable Alkaloids. *American Journal Medical Sciences*. October, 1862. 28 pages.

20. Discovery of Cholesterine and Seroline as secretions in health of the Salivary, Tear, Mammary and Sudorific Glands; of the Testis and Ovary; of the Kidneys in Hepatic Derangements; of Mucous Membranes when congested and inflamed, and the fluids of Ascites and that of Spina Bifida. Do., April, 1863. 2 plates. 17 pages.

21. Remarks on Fungi, with an Account of Experiments showing the influence of the Fungi of Wheat and Rye Straw on the Human System, and some observations which point to them as the probable source of Camp Measles, and perhaps of Measles generally. Do., July, 1862. 1 plate. 20 pages.

22. Inoculating the Human System with Straw Fungi to protect it against contagion of Measles, with some additional observations relating to the influence of Fungoid growths in producing disease, and in the Fermentation and Putrefaction of Organic Bodies. Do., October, 1862. 8 pages.

23. Parasitic Forms Developed in Parent Epithelial Cells of the Urinary and Genital Organs, and in the Secretions. With 34 illustrations. Do. April, 1868.

24. Remarks on the Structure, Functions and Classification of the Parent Gland Cells, with Miscroscopic Investigations relative to the causes of the several varieties of Rheumatism, and directions for their treatment. 1 plate of illustrations. Do., October, 1867. 19 pages.

25. Microscopic Researches relating to the Histology and Minute Anatomy of the Spleen and Lacteal and Lymphatic Glands, showing their ultimate structure and their organic elements, of their highly interesting and important functions, with some remarks on the cause of ropiness of Mucus and the tendency of all healthy and many diseased cells to be metamorphosed into filaments. 1 plate. 34 pages. Do., April, 1866.

26. Description of two new Algoid Vegetations, one of which appears to be the specific cause of Syphilis and the other of Gonorrhœa. With 16 illustrations. Do., 1867. Also *Zeitschrift für Parasitenkunde*. 1878.

27. Geological Report of the Mill Creek Canal Coal Field. With one map and two plates. Published in Cincinnati, 1859.

28. Analysis, Organic and Inorganic, of the Cucumber. *Cultivator*. 1849.

28. Experiments on the Capillary Attractions of the Soil, explaining some important and interesting principles and phenomena in Agriculture and Geology. *The American Polytechnic Journal*. 1853.

30. A New Carbonic Acid Apparatus. Do., 1853.

31. Analysis of Dead Sea Water. 1854.

32. Two Interesting Parasitic Diseases; one we take from sucking kittens and the other from sucking puppies—Trichosis Felinus and T. Caninus. *Boston Medical and Surgical Journal*, June 4th, 1868. 6 illustrations. Also, *Zeitschrift für Parasitenkunde*, Hallier. Jena., 1875.

33. Pus. and Infection. *Boston Journal of Chemistry.* January, 1878.
34. Microscopic Examinations of Blood and the Vegetations found in Variola, Vaccine and Typhoid Fever. 66 pages and 62 illustrations. Published by Moorehead, Bond & Co., New York. 1868.
35. Vegetations found in the Blood of Patients Suffering from Erysipelas. *Halliers Zeitschrift für Parasitenkunde.* 1873. 8 illustrations.
36. Infusorial Catarrh and Asthma. 18 illustrations. Do., 1873.
37. Analysis, Organic and Inorganic, of the White Sugar Beet. The *Albany Cultivator.* October, 1851.
38. Analysis, Organic and Inorganic, of the Parsnip. N. Y. State Agricultural Report. 1851.
39. Ancient Rock and Earth Writing and Inscriptions of the Moundbuilders, with a description of their fortifications, enclosures, mounds and other earth and rock works. 49 plates. In the hands of the American Antiquarian Society, and only partly published in their transactions and in the Ohio Centennial Report. 1863.
40. Influence of the Position of the Body upon the Heart's Action *American Journal Medical Sciences.* 1865.
41. Material Application of Chemistry to Agriculture. The *Albany Cultivator.* 1851.
42. Analysis, Organic and Inorganic, of the several kinds of Grains and Vegetables. The *Albany Cultivator.* August, 1849.

UNPUBLISHED WORKS AND PAPERS.

1. Diphtheria, its Cause and Treatment. 3 plates of illustrations. 1862.
2. Asthma, the various forms of, and their causes and treatment. 3 plates of illustrations. Ready for press since 1866.
3. Consumption, its Causes and Treatment. 4 plates. Ready for press in 1867.
4. Hog Cholera, its Cause and Prevention. 1858.
5. Ultimate Structure and Functions of the Liver. 1865. 3 plates.
6. Ultimate Structure and Functions of the Kidneys. 1864. 2 plates.
7. Geological Report of the Coal Fields of Virginia and Kentucky. 1857. With maps and many illustrations.
8. Histology of Plants. Prize Essay. 65 illustrations. 1848.
9. Causes and Treatment of "Bright's Disease." 1865.
10. Causes and Treatment of Diabetes. 1864.
11. Causes and Treatment of Goitre, Cretinism, Ovarian Tumors and other Colloid Diseases. 1863.
12. Causes and Treatment of Progressive Locomotor Ataxy. 1867.
13. Cause and Treatment of Fatty Diseases of the Heart, Liver and Spleen. 1864.
14. Cause and Treatment of Paresis. 1865.
15. One of the Most Common Causes of Paralysis, with Treatment. 1867.
16. Microscopic Examinations connected with Spermatozoa and Ova, with contents of Pollen Grains and Modes of Development of Zoosporoid Cells. 1860.

17. Cryptogamic Spores in the Tissues of the Living Animal. Their development in food one source of disease, and a cause of fermentation, gangrene or death and decay in organized bodies. 7 plates and 102 illustrations.

18. Microscopic Investigations connected with the Exudation and Expectoration of Angina membranacæ and Gangrenosa and Scarlatina Anginosa, resulting in the discovery of their true source, and the pathological process by which the exudations are produced; and the further discovery of a peculiar fungus belonging to the Genus Peronospora, developing in the sloughs and membranes, the spores of which are infectious and produce the disease; also some general conclusions on the Ætiology of Fevers, the peculiar functions of the Epithelial cell envelope, and the probable way in which the system receives a more or less permanent protective immunity by one attack of certain contagious diseases against a second invasion of the same. 3 plates. 160 illustrations. 1862.

19. Description of several new species of Ascaridæ found on and in the human body, and a brief account of several new Entozoa. 2 plates and 30 figures. 1865.

20. Investigations connected with the Cause and Treatment of Paralysis of the Will, Paralysis of the Memory and Paralysis of the entire Intellectual and Moral Faculties, causing a peculiar mental state and Insanity.

Dr. Salisbury regards the vegetation in syphilis as a true taint, since it will grow in any one to whom it is communicated by infection. The taint in consumption is not so readily imparted by infection. It is made up of vegetative spores of a yeast in the blood, not exactly the ordinary domestic yeast, but a saccharine, acid, fermentative vegetation that thrives on starch and sugar. It is found in the blood of the parent. It is found in the blood of the child. It exists in a latent state and is found also one year before organic lung disease. I have seen Dr. Salisbury's records of the autopsies of 104 frogs killed by inducing consumption in them artificially. (Ready for the press in 1867.) I call this the synthesis of the disease. Also I show you bread raised with the yeast found in the diarrhœal dejections of a third stage consumptive, made at Dr. Salisbury's suggestion, and following the same course he pursued years ago. He was unable to raise bread with healthy fecal dejections. I am now engaged in the effort to publish Dr. Salisbury's works on the causes and treatment of consumption based on 1,000 cases, ready for the press in 1867, corroborated by me. Fasciculus I, A New Physical Sign of the Pretubercular State ; II, Morphology of Consumptive Blood ; III, Treatment on the Salisbury Plan. About 1,500 page of manuscript fully illustrated. In this work will be found the fullest account of the author's views on the subject of tubercle. Upon consultation it has been deemed best to publish it by subscription, and my effort is to secure 1,000 subscriptions. I respectfully ask your encouragement in this undertaking.

Your attention is now directed to the question 1, What is yeast? This is a cryptogamic vegetation, well known to man from the earliest ages of antiquity, and now of common use in every household, growing concealed in the darkness where there are warmth and moisture, a plant that is little larger than a red blood corpuscle; a plant that produces alcohol, that has a kinetic energy of 200 lbs. to the square inch; one that is a protoplasm, and that thus furnishes a clear illustration of a fungus and of what the author regards as an hereditary taint. We will show these and a few other fungi, some of which are nocent and some innocent. 2. I show a few algae, some nocent and some innocent, in order to exhibit a genus of parasitic vegetations that includes 20,000 species—(fungi, 8,000, Reinsch)—so that you may see what they resemble, and may realize that the opposition to the germ theory of disease is fallacious, since it is based upon the idea that because so many fungi are innocent, therefore they *all are.* Mushrooms are fungi, and people have been known to die, poisoned by eating them. 3. I show what blood is morphologically in healthy men, and in some animals. 4. The form element found in syphilitic blood, constituting the taint. 5. Consumptive blood taints. 6. Two photographs showing the physical effects on the blood of the Salisbury plan of treatment. 7. Sources of error.

List of microphotographs shown, most of them taken with Tolles' objectives.

1. Microphotographic apparatus of the writer.

2. Vaccine virus, 1-10th inch objective, to illustrate Beale's doctrine.

3, 4, 5, 6, 7, 8, 9. Yeast plants, spores, barley, starch, grain, 1-50th. (For the sake of brevity the words "inch, objective," are hereafter omitted.)

10. Budding of yeast. After Mitscherlich diagram.

11, 12. Mycelial filaments found in connection with yeast; aërial, 1-10.

13. Yeast sporangia, 1-75.

14. Butyric acid, fermentative vegetation, (rancid butter), 1-10.

15. Lard, 1-10.

16. Sewer gas fermentative vegetation, 1-10.

17. Diphtheritic membrane kept 3½ years, in full strength carbolic acid, 1-10.

18. Algae. Leptothrix buccalis from mouths, innocent, ¼.

19. Algae infesting other algae, some nocent and some innocent. After Reinsch.

20. Innocent parasitic algae in a Key West sea weed, Reinsch, ¼.

21. Cosmarium binoculatum infested by an alga that kills its hosts. Cochituate water, 1-10, R.

22. Healthy blood, horse, 1-50.

23. Healthy blood, man's, living white corpuscle, 1-50.

24, 25, 26. Amœboid movement of white blood corpuscle—photos. of drawings made from images thrown upon a screen with the sun, from life, 1-5.

27. Human red blood corpuscles, dry, 1-75.

28. Rat's blood, dead, 1-50.

29. Ox, alive, 1-50.

30. Horse, 1-50, alive.

31. Rabbit dead and frozen, 1-50.

32. Sheep, alive, 1-50.

33. Deer dead and frozen, 1-50.

34. Dog, alive, 1-50.

35. Mouse, dead, 1-50.

36. Trout dead and frozen. The last nine were taken at the same amplification for medico-legal comparison.

37. Blue bird's blood, 1-16.

39. Frog's blood, 1-50.

40. Blood filled with spores that may be syphilitic or consumptive, distinguished under the microscope (not in photo.) by the copper color, 1-10. Wet.

41. 3 white corpuscles, syphilis—enlarged—wet, ¼.

42. Mycelial filaments, syphilis—wet, ¼ enlarged.
43. " " " dry, 1-50.
44. " " " wet, 1.16.
45. " " " enlarged.
46. White and red corpuscles, syphilis, 1-50.
47, 48. White and red corpuscles, syphilis, 1-75.
49. Pretubercular blood showing spores—an hereditary taint —found one year before organic lung disease, wet.
50. Collection of mycelial filaments, consumptive, 1-5. Blood dried under the cover.
51. Consumptive blood drying under cover, vacuolated, shows the futility of taking blood home for examination after several days.
52. Mycelial filament, consumptive blood, wet, ¼ enlarged.
53. Dried mycelial filaments traversing an air bubble—wet and drying up, 1-10.
54. White blood corpuscles, consumptive, 1-50.
54. 4 " " " 1-50.
56. 3 " " " 1-75. First photo. taken with this objective.
57. White blood corpuscle showing entophytal growth, consumptive, 1-50, certified to by Prof. Reinsch.
58. Fibrin filaments, 1-16 wet, consumptive. Foreign substances liable to be mixed with the blood.
59. Hair, 1-10 ; consumptive, wet.
60. Epithelial scale from the skin, 1-10 wet.
61. Cotton fiber fibrillated, wet, 1-10.
62. Dust from furniture, wet, 1-10.
63. Common toilet soap, 1-10.
64. Typical 3d stage consumptive blood, 1-16, dry.
65. " " " " three months on the Salisbury plan, 1-16, dry,

In order to do justice to my associate, allow me to say that this microphotographic work was done with the assistance of Dr. G. B. Harriman, dentist, of Boston, the owner of the 1-50 and 1-75 inch objective, which he has allowed me to bring. He is also the discoverer of fibers in Dentine (*American Journal of Dental*

*Science*, May, 1870), and of nerve fibers in the soft solids of the Dentine (*Dental Cosmos*, January, 1870.)

The following extract from the *Journal de Micrographie*, Paris, November, 1877, is the concluding portion of a notice by Dr. Pelleton of the work done by Dr. Harriman and other microphotographers of Boston. Some of the specimens exhibited to you this evening were included in the collection to which Dr. Pelleton refers:

"To sum up, these microphotographs, which we think ought to interest our readers for a long time, because of the considerable interest which the photographic reproduction of microscopic objects presents are remarkable; and so much the more as all have been executed by, except, of course, those who transferred the impression to the stereotype plate, doctors, who are amateurs, and not by professional photographers. The work is of a nature to give a high idea of the objectives with which the views have been obtained; and we can compare them only to the best photographs executed by Dr. Woodward with the ⅛ and 21-16 inch objectives of Powell and Lealand, or by some European operators with obectives of Hartnack and Prazmonski; but if we think we have seen some which are as remarkable, we do not think we have encountered any that are their superiors."

It remains for me to thank you for your fair hearing. I do not ask your endorsement of my views. It took three years to satisfy myself. I hope it will not take you so long; nor do I hope you will dismiss the question at once by declaring the whole matter visionary—a matter of mere words—the vagaries of an enthusiastic microscopist. On general principles, those who study a subject most, are usually conceded to know the most about it. Dr. Salisbury began in 1857, I in 1868; hence it is hoped that some of your careful observers may be induced to go over the ground for themselves, carefully, in actual cases—not deciding till they have seen the work here discussed, as of course a public presentation like this must necessarily pass by many important points. Allow me simply to name a few essentials: 1. The blood must be inspected in the shortest possible space of time after withdrawal. (I have invented a machine to get a drop of blood more quickly.) 2. The objective must be good as to color and definition. Such an one can be obtained of the Boston Optical Works—Tolles', third class, 1-5 inch, with clinical stand (suggested by me) for $25. This instrument is portable and can be used with any first class objective. (The photos. of yeast, 1-50 inch objective, were taken with this clinical stand.) Ten minutes object teaching suffices to get an idea of this simple instrument. Further, in connection with my associates, I am preparing a clinical microscopic primer for the use of those who are willing to learn. I am glad to say to the older men, who relegate such work to young men, that, strange though it seem, microscopy is a solace of old age. Shrewbury worked with it till his death at about 90 years. A good microscope strengthens the eyes, in place of weakening them.

Finally, I may be mistaken, and past experience may not be realized in the future; but I think that should Dr. Salisbury's and my own experience be repeated (I do not say it will be) by all physicians in the United States, 13,000 lives could be annually saved by the detection of the taints of the pretubercular state alone. I believe it a duty to promulgate these views. I should like to see published the full text, of which but a few pages have been given. I desire that some influential authoritative body appoint a commission of 100 medical men of commanding character. Let them send patients to Dr. Salisbury and myself, or some one else, for one year's treatment on the Salisbury plan, the records to be kept, blood to be photographed and information to be from time to time given to the committee of 100, without discussion or controversy. At the year's end let the authoritative body summon the committee of 100 in council. By its decision after faithful work we would abide. The John Hopkins foundation could furnish such an opportunity. Dr. Salisbury wants such a test. I am willing to conduct it.

Gentlemen, if you can express a favorable opinion on the work of this evening, please to say that you approve of such a test in Baltimore.

CHICAGO, February 17th, 1879.

